# Editorial: The Genetic and Epigenetic Bases of Cellular Response to Ionizing Radiation

**DOI:** 10.3389/fgene.2022.857168

**Published:** 2022-03-04

**Authors:** Ki Moon Seong, Giovanni Cenci

**Affiliations:** ^1^ Laboratory of Biological Dosimetry, National Radiation Emergency Medical Center, Korea Institute of Radiological and Medical Sciences, Seoul, South Korea; ^2^ Dipartimento di Biologia e Biotecnologie “C. Darwin”, Sapienza Università di Roma, Rome, Italy; ^3^ Fondazione Cenci Bolognetti/Istituto Pasteur Italia, Rome, Italy

**Keywords:** genetic alteration, epigenetic changes, cellular response, ionizing radiation, reactive oxy gen species

**FIGURE 1:**
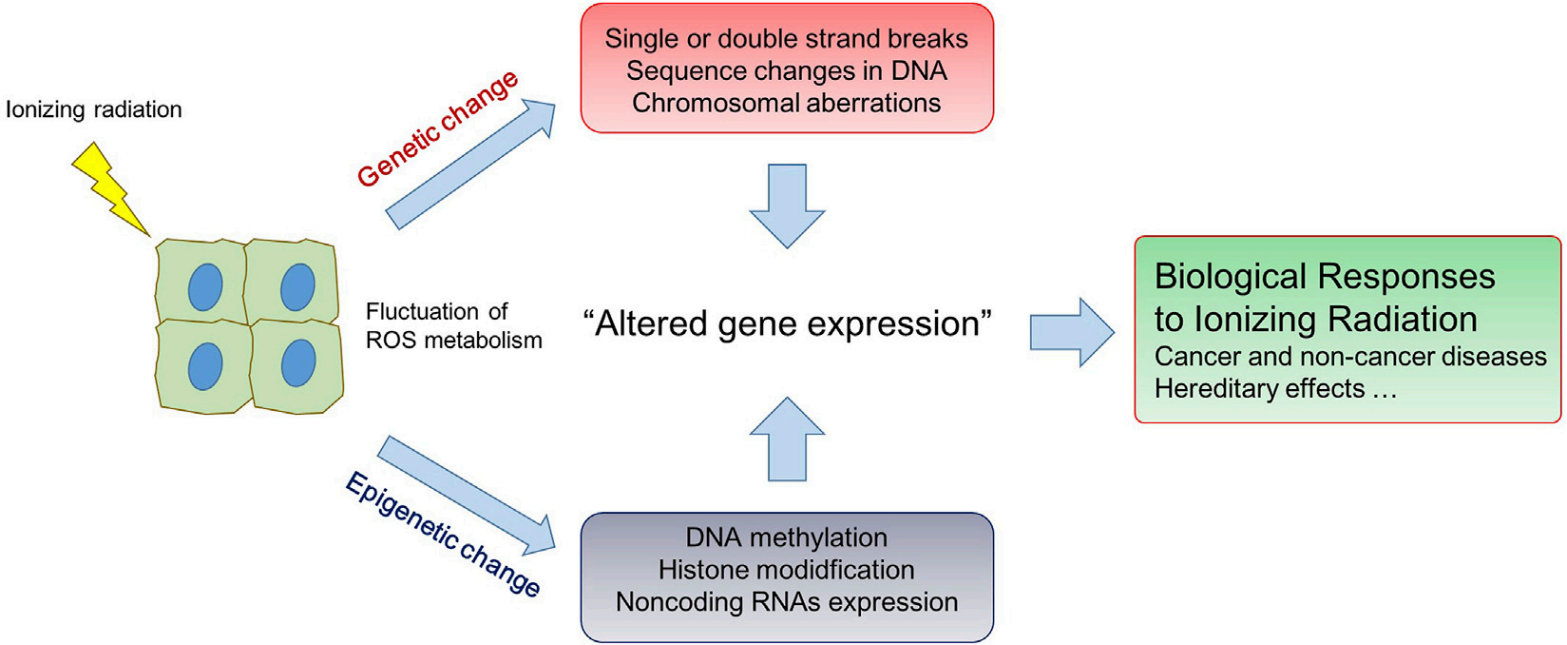
Genetic and epigenetic change of cells responding to ionizing radiation.

Since the discovery of X-ray in 1895, ionizing radiation has been well known to be one of genotoxic stresses which, by direct deposition of energy and by indirect action of reactive oxygen species, can induce a wide spectrum of DNA alterations including structural changes of DNA molecules, sequence mutation, and consequently chromosomal aberrations (Goodhead, 1994; Nikjoo et al., 2001). Depending on the dose and/or dose rate, radiation-induced DNA lesions could lead to detrimental effects on the biological organism from unicellular systems to human. Exposure to high dose radiation (HDR) is indeed a public concern as, leading to tissue damage and ultimately cancers, represents a great health risk for humans. Recently, the attention of radiation exposure has moved to low dose radiation (LDR; less than tens of millisieverts), to which people are often exposed in daily life such as during medical, occupational and environmental exposure. Accumulated evidence described that LDR could control immune system accompanying with accumulation of DNA damages and oxidative stress and even benefited the viability in some organs (Shin et al.). However, biological effects of LDR are not fully understood and could not be concluded as harmful to human since there are some uncertainties in the risk assessment. A great number of works demonstrated that radiation could induce epigenetic effects (i.e., DNA methylation, histone modifications, and expression of non-coding (nc) RNAs) which can change gene expression without altering DNA sequences. Since epigenetic effects are susceptible to environmental changes, they are thus considered to be a possible mechanism to explain the long-term effects after radiation exposure (Ilnytskyy and Kovalchuk, 2011; Miousse et al., 2015; Horemans et al., 2019). Understanding genetic and epigenetic responses of ionizing radiation is paramount to assess health risks for radiation exposure, especially long-term exposure with low level (Figure 1). The fluctuation of reactive oxygen species (ROS) metabolism, which can mediate the alteration of gene expression by both genetic and epigenetic changes, is an important physiological aspect to take into account when considering radiation responses (Jansen et al., 2021). Radiation-induced ROS generation or removal is involved in the cell deaths and adaptive responses of many cell types, including mesenchymal stem cells (MSCs) which is highly valuable in the medical application of MSC (Konkova et al.).

Changes in DNA methylation, the addition of methyl group at CpG sites of DNA backbone (5-metylcytosine), can explain the radiation sensitivity in a manner that depends on the exposed doses-, sex-, tissue-specificity (Pogribny et al., 2004; Raiche et al., 2004). Differential epigenetic changes caused by radiation exposure correlated with cell cycle, DNA repair, and apoptosis (Antwih et al., 2013). Histone modification such as phosphorylation, methylation, and acetylation is well known to play a key role in DNA repair mechanism responding to radiation exposure (Di Nisio et al., 2021). In addition, epigenetic changes regulated by radiation exposure can be governed by the mobilization of Transposon Elements (Yushkova). It can ultimately influence the inheritance of radiation-induced instability, interplaying among the transposon and crucial DNA repair genes after chronic radiation exposure (Yushkova and Bashlykova, 2021). Finally, radiation exposure affects the regulation of ncRNAs especially microRNAs (miRNAs) that play specific roles in controlling gene expression. Radiation-induced miRNAs have shown to have a fundamental role in the many physiological pathways determining the fate of cells, including cell cycle, cell death, and modulation of cells function (Chaudhry et al., 2010).

Considering environmental factors such as the ethnicity and natural background level in the radiation mechanism of biological effects can also reduce the uncertainties in the risk assessment on long-term exposure to LDR. Ethnicity in different living environments can determine different cytogenetic responses against radiation-induced genotoxic stress at the level of human populations (Soumboundou et al.). It should be taken in great consideration when adequate biological dosimetry and radiation therapy is required in cancer patients. Many studies reported that people in high level background radiation area above 10 mSv/year showed no health risks for cancers or life-shortening, compared to the residents in other areas (Hendry et al., 2009). Furthermore, underground biology studies in Deep Underground Laboratories (DULs) characterized by substantial reduction of the exposure to environmental radiation background showed that background level of radiation could influence the sustaining the life of organisms, modulating the expression of several genes involved in protein metabolism and stress response (Esposito et al., 2020; Castillo et al.).

The articles of this research topic provide valuable information on the genetic and epigenetic modification responding to radiation exposure in bacteria, and eukaryotic organisms including humans. These findings will contribute to more comprehensive understanding for cellular response to radiation and shed light on the future studies of health risk assessment for radiation exposure.
